# Import and Export of Mannosylerythritol Lipids by Ustilago maydis

**DOI:** 10.1128/mbio.02123-22

**Published:** 2022-09-07

**Authors:** Fabienne Becker, Uwe Linne, Xiulan Xie, Anna-Lena Hemer, Michael Bölker, Johannes Freitag, Björn Sandrock

**Affiliations:** a Department of Biology, Philipps University Marburg, Marburg, Germany; b Department of Chemistry, Philipps University Marburg, Marburg, Germany; c SYNMIKRO Center for Synthetic Microbiology, Philipps University Marburg, Marburg, Germany; Leibniz-HKI and University of Jena; University of Minnesota Medical School

**Keywords:** secondary metabolites, glycolipids, mannosylerythritol lipids, major facilitator transporter, *Ustilago maydis*

## Abstract

Upon nitrogen starvation, the basidiomycete Ustilago maydis, which causes smut disease on corn, secretes amphipathic glycolipids, including mannosylerythritol lipids (MELs). MELs consist of a carbohydrate core whose mannosyl moiety is both acylated with fatty acids of different lengths and acetylated. Here, we report the transport of MELs into and out of the cell depending on the transport protein Mmf1, which belongs to the major facilitator superfamily. Analysis of *mmf1* mutants and mutants lacking the acetyltransferase Mat1 revealed that Mmf1 is necessary for the export of acetylated MELs, while MELs without an acetyl group are secreted independently of this transporter. Upon deletion of *mmf1*, we detected novel MEL species lacking the acyl side chain at C-3′. With the help of feeding experiments, we demonstrate that MELs are taken up by U. maydis in an *mmf1*-independent manner. This leads to catabolism or rearrangement of acetyl and acyl side groups and subsequent secretion. The catabolism of MELs involves the presence of Mac2, an enzyme required for MEL biosynthesis. In cocultivation experiments, mutual exchange of MELs between different mutants was observed. Thus, we propose a novel function for fungal glycolipids as an external carbon storage.

## INTRODUCTION

Fungi produce and secrete a large variety of chemically distinct molecules known as secondary metabolites (1). They fulfill very different biological functions, e.g., protection from sunlight, defense against competitors, and developmental signaling (1, 2). Genes coding for enzymes and auxiliary factors for secondary metabolite production are often arranged as gene clusters and are usually coregulated (1).

Two different types of glycolipids, mannosylerythritol lipids (MELs) and ustilagic acids (UAs), belong to the secondary metabolites produced by the basidiomycetous fungus Ustilago maydis and some of its close relatives. They act as biosurfactants and exhibit antimicrobial activity (3–5). MELs are a valuable natural resource for biotechnology and pharmaceutical products and have the potential to replace mineral-oil-based surfactants in many applications (6–9).

**FIG 1:**
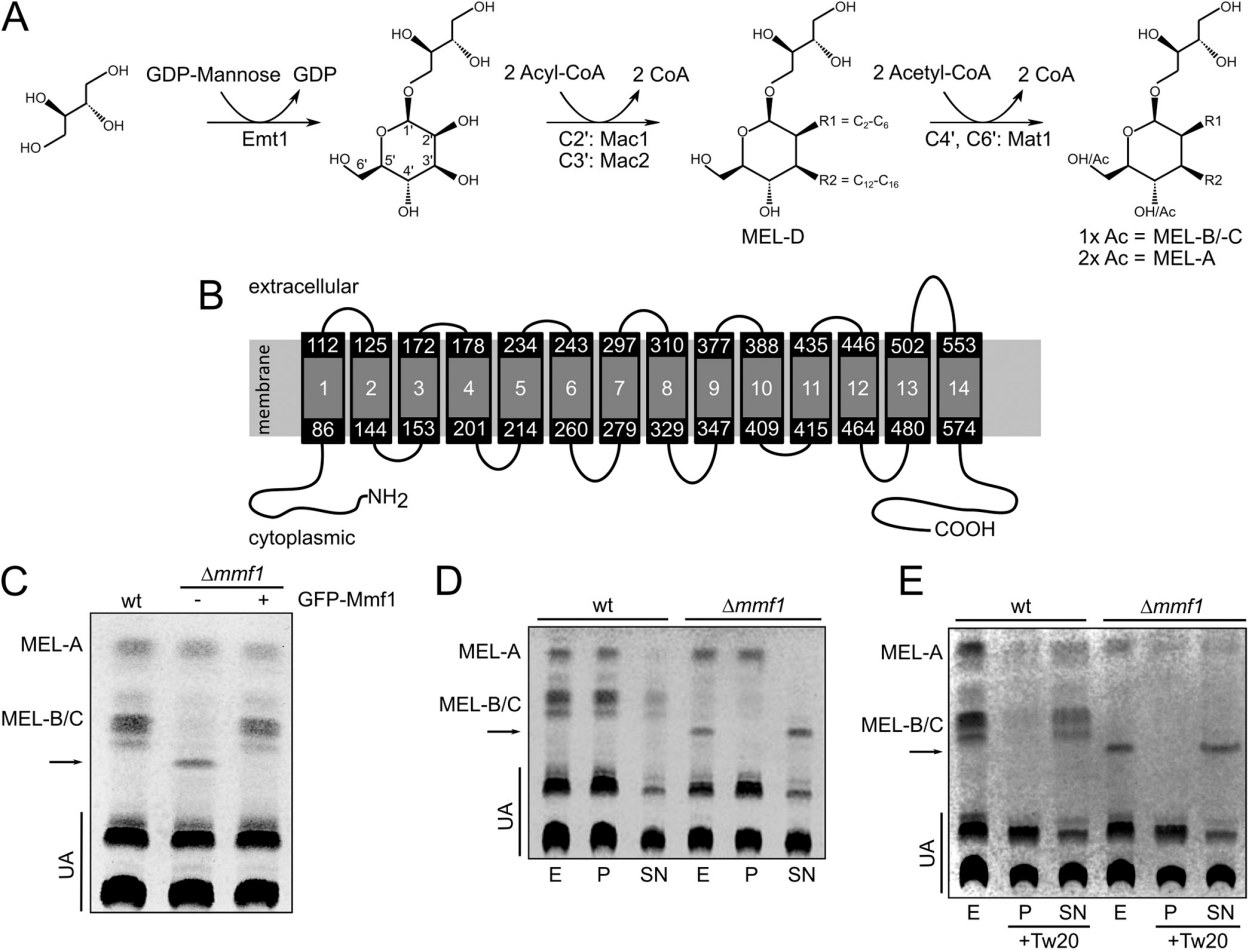
Mmf1 specifically exports acetylated MELs. (A) MEL biosynthesis pathway. C-2′ and C-3′, positions of acylation of the mannose moiety by Mac1 and Mac2, respectively; C-4′ and C-6′, positions of acetylation (Ac) of the mannose moiety by Mat1; R1 and R2, fatty acid side chains of the indicated lengths. (B) Topology of U. maydis Mmf1 according to Phyre2 prediction (53). Depicted are 14 transmembrane domains and their potential orientation within the plasma membrane. (C) TLC showing glycolipids prepared from wild-type cells, Δ*mmf1* cells, and Δ*mmf1* cells expressing GFP-Mmf1. The arrow highlights a novel MEL variant. UA, ustilagic acid. (D) The indicated cultures producing glycolipids were fractionated. Cells of the indicated strains were pelleted, the supernatants were saved, and the pellets were washed two times with fresh medium. The supernatants were centrifuged at high speed to remove residual cell material. The resuspended pellets (P) and the supernatants (SN) were extracted with ethyl acetate and analyzed by TLC. As control the entire culture was extracted (E). (E) Experiment performed as described above for panel D but with 0.1% Tween 20 (Tw20) added to the culture before the initial centrifugation step to separate the pellet and supernatant.

Genes required for MEL biosynthesis are organized in a gene cluster, which encodes four enzymes specifically involved in MEL production and one transport protein of the major facilitator superfamily (MFS) termed Mmf1 (see Fig. S1A in the supplemental material). The expression of cluster genes is highly induced upon nitrogen starvation (10). The glycosyltransferase Emt1 is necessary for the synthesis of the hydrophilic carbohydrate backbone 4-*O*-β-d-mannopyranosyl-erythritol (Fig. 1A). The two acyltransferases Mac1 and Mac2 are peroxisomal enzymes coupling the acylation of MELs to the β-oxidation of fatty acids (10, 11). The acetyltransferase Mat1 is localized at the cellular membrane and catalyzes the formation of differentially acetylated MEL variants termed MEL-A, MEL-B, and MEL-C (Fig. 1A). In the absence of Mat1, only the nonacetylated variant MEL-D is released (10). Several other related basidiomycetes contain similar gene clusters to facilitate MEL synthesis (12–16).

10.1128/mbio.02123-22.1 FIG S1 (A) MEL gene cluster at the chromosomal end of chromosome 7. (B) Phylogeny of Mmf1 with putative Mmf1 transporters from other fungi. The best hits of Mmf1 in the proteomes of Caenorhabditis elegans and Zea mays are also shown. An ortholog of the MEL transporter could not be identified in humans or higher eukaryotes. Download FIG S1, PDF file, 0.2 MB. Copyright © 2022 Becker et al. 2022 Becker et al. https://creativecommons.org/licenses/by/4.0/ This content is distributed under the terms of the Creative Commons Attribution 4.0 International license.

Transport proteins of different families are often encoded by gene clusters for glycolipids to regulate their export (17, 18). Major facilitator superfamily transport proteins are ubiquitous in all organisms and contain 12 or 14 transmembrane-spanning domains (19). MFS proteins are involved in the transport of ions but also larger molecules across membranes. Mutations in several of these proteins are linked to human diseases like Alzheimer’s disease, cancer, or schizophrenia (19). In U. maydis, 91 genes encoding MFS proteins have been identified (20). Three of them are part of characterized gene clusters involved in the production of the secondary metabolites itaconic acid (Itp1), siderophores (Fer7), and MELs, but their molecular function remained elusive (10, 21, 22). Two other MFS transporters, Srt1 and Hxt1, are involved in sugar transport during biotrophic development of U. maydis (23, 24). Recently, it was shown that in the related fungus Pseudozyma tsukubaensis, the deletion of a gene encoding the homolog of Mmf1 leads to the secretion of unusual MELs that harbor only a single acyl group (25).

Although many aspects of MEL biosynthesis are well characterized, their biological function and transport routes remain elusive. Here, we show that U. maydis cells can import MELs, leading to their modification or metabolism. Hence, MELs may act as an external carbon storage and supply. We also demonstrate that the transport protein Mmf1 specifically exports acetylated MELs.

## RESULTS

### Mutants lacking the transport protein Mmf1 fail to secrete acetylated MELs.

To address the route of transport of MELs in Ustilago maydis, we first investigated the function of the membrane protein Mmf1 encoded in the gene cluster for MEL biosynthesis (see Fig. S1 in the supplemental material). Mmf1 contains 14 putative transmembrane domains and belongs to the major facilitator family (Fig. 1B and Fig. S1B). We deleted *mmf1* in the genome of U. maydis and analyzed MELs from total cell extracts by thin-layer chromatography (TLC). The deletion strain showed a reduced total amount of MELs and produced a novel MEL variant with altered hydrophobicity not observed in wild-type (WT) strains (Fig. 1C). In addition, small amounts of diacetylated MELs were detected. The amounts of monoacetylated MEL-B and MEL-C were drastically reduced (Fig. 1C). The phenotype of the Δ*mmf1* mutant was complemented upon the introduction of a construct encoding a wild-type version of the gene fused to the C-terminus of green fluorescent protein (GFP) (Fig. 1C). We next examined if the subcellular localization of enzymes involved in MEL production changes upon deletion of *mmf1*. The localization of GFP-Mac2 (peroxisomes), GFP-Mat1 (cell periphery), and GFP-Mmf1 (plasma membrane) remained unchanged in Δ*mmf1* cells, suggesting that the intracellular localization of MEL biosynthesis is not perturbed in this mutant (Fig. S2A to C) (11).

10.1128/mbio.02123-22.2 FIG S2 GFP-tagged versions of Mac2 (A), Mat1 (B), and Mmf1 (C) in the indicated strains were analyzed by fluorescence microscopy. The fungal cell wall was stained with calcofluor white (CFW). Bars, 5 μm. Download FIG S2, PDF file, 0.9 MB. Copyright © 2022 Becker et al. 2022 Becker et al. https://creativecommons.org/licenses/by/4.0/ This content is distributed under the terms of the Creative Commons Attribution 4.0 International license.

We hypothesized that the phenotype of the Δ*mmf1* mutant might result from the decreased secretion of acetylated MELs, which could be specific substrates of the putative transporter localized in the plasma membrane (Fig. S2C) (11). Impaired secretion of MELs may trigger their degradation or modification in the cytosol. First, we tested for the secretion of MELs by analyzing glycolipids from the cell pellet and supernatant fractions. Di- and monoacetylated MELs were mainly part of the pellet fraction in WT and Δ*mmf1* mutant cells, whereas the MEL variant specific for the Δ*mmf1* mutant was completely soluble (Fig. 1D). It is known from previous work that U. maydis glycolipids (ustilagic acid and MELs) precipitate as needle-like structures upon secretion (26). To improve detection of MELs secreted into the supernatant, the nonionic detergent Tween 20 was added before extraction from the pellet and supernatant fractions. Both mono- and diacetylated MELs were almost entirely secreted in wild-type cells (Fig. 1E), indicating a role of Mmf1 in the export of mono- and diacetylated MELs. This may explain the absence of monoacetylated MELs in Δ*mmf1* cells. If these fail to be secreted, they are likely to be converted into diacetylated MELs in the cytosol prior to degradation.

In a complementary approach to improve the detection of secreted MELs, we aimed to eliminate coprecipitation of secreted MELs and ustilagic acids. To circumvent the formation of these precipitates, we analyzed the secretion of MELs in Δ*rua1* and Δ*rua1* Δ*mmf1* strains. Rua1 is a transcription factor that regulates the formation of ustilagic acids, the major constituent of the needle-like structures (27, 28). Indeed, in cultures of Δ*rua1* cells, acetylated MELs predominantly occurred in the supernatant and did not precipitate with the cells. Δ*rua1* Δ*mmf1* double mutants did not form large amounts of acetylated MELs, and only the unusual hydrophilic MEL species was found in the supernatant (Fig. S3). Together, these data suggest a specific function of Mmf1 as exporter of mono- and diacetylated molecules.

10.1128/mbio.02123-22.3 FIG S3 Secretion of MELs in the absence of ustilagic acids. Cells lacking UA biosynthesis (Δ*rua1*) were analyzed as described in the legend of Fig. 1D. Download FIG S3, PDF file, 0.9 MB. Copyright © 2022 Becker et al. 2022 Becker et al. https://creativecommons.org/licenses/by/4.0/ This content is distributed under the terms of the Creative Commons Attribution 4.0 International license.

### The unusual MEL species is derived from acetylated MEL-A.

**FIG 2:**
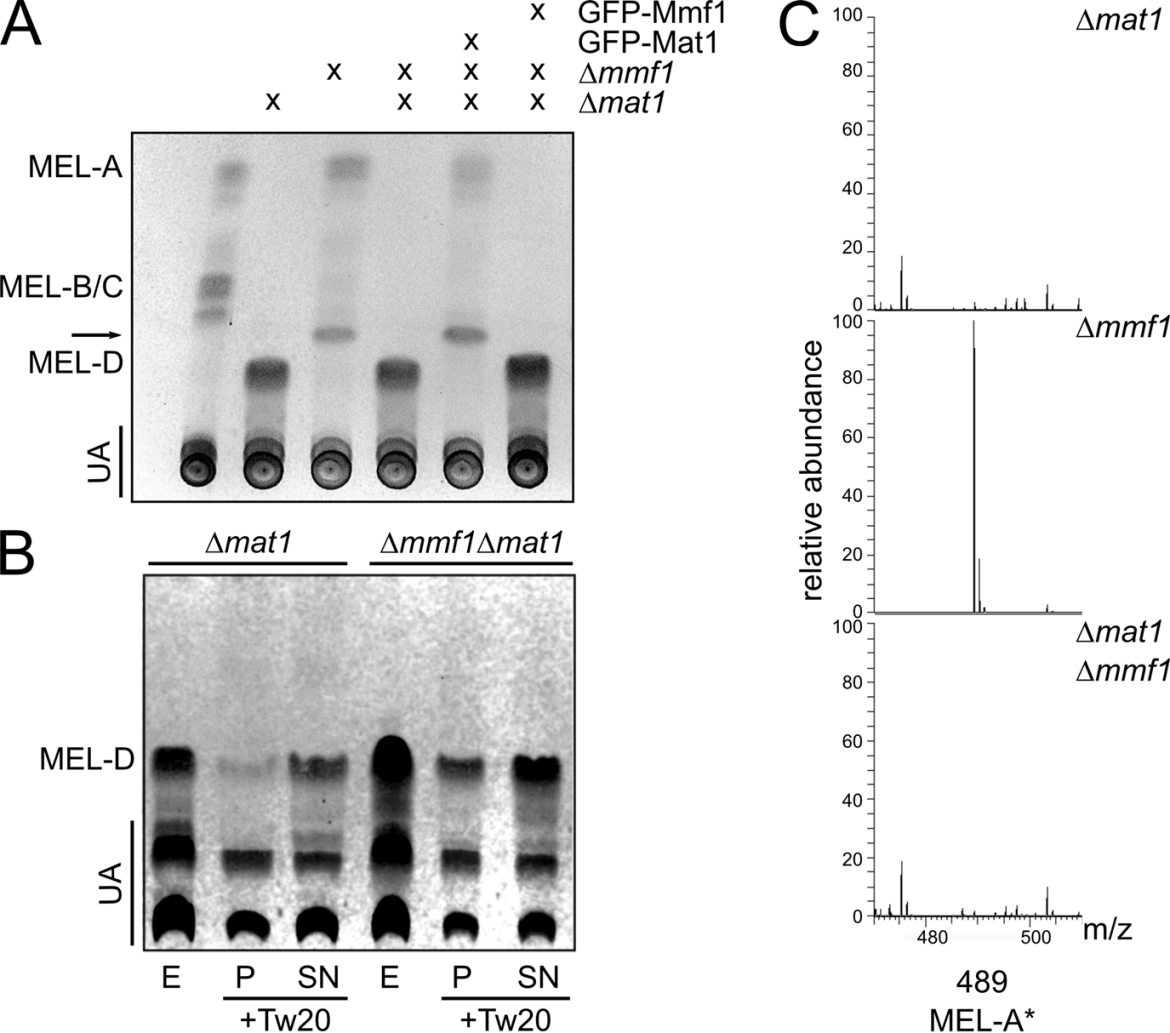
Mmf1 is dispensable for the secretion of nonacetylated MELs. (A) TLC of glycolipids produced by the indicated strains. The arrow marks the novel MEL-A* variant. (B) The indicated cultures producing glycolipids were fractionated, and 0.1% Tween 20 (Tw20) was added to the culture before the initial centrifugation step to separate the pellet (P) and the supernatant (SN). Glycolipids were prepared as described in the legend of Fig. 1E. (C) Total ion counts of MELs from the indicated strains analyzed by liquid chromatography-mass spectrometry (LCMS). Peak 489 specifies the mass of the novel MEL-A* variant of 489.1924 *m/z*.

**FIG 3:**
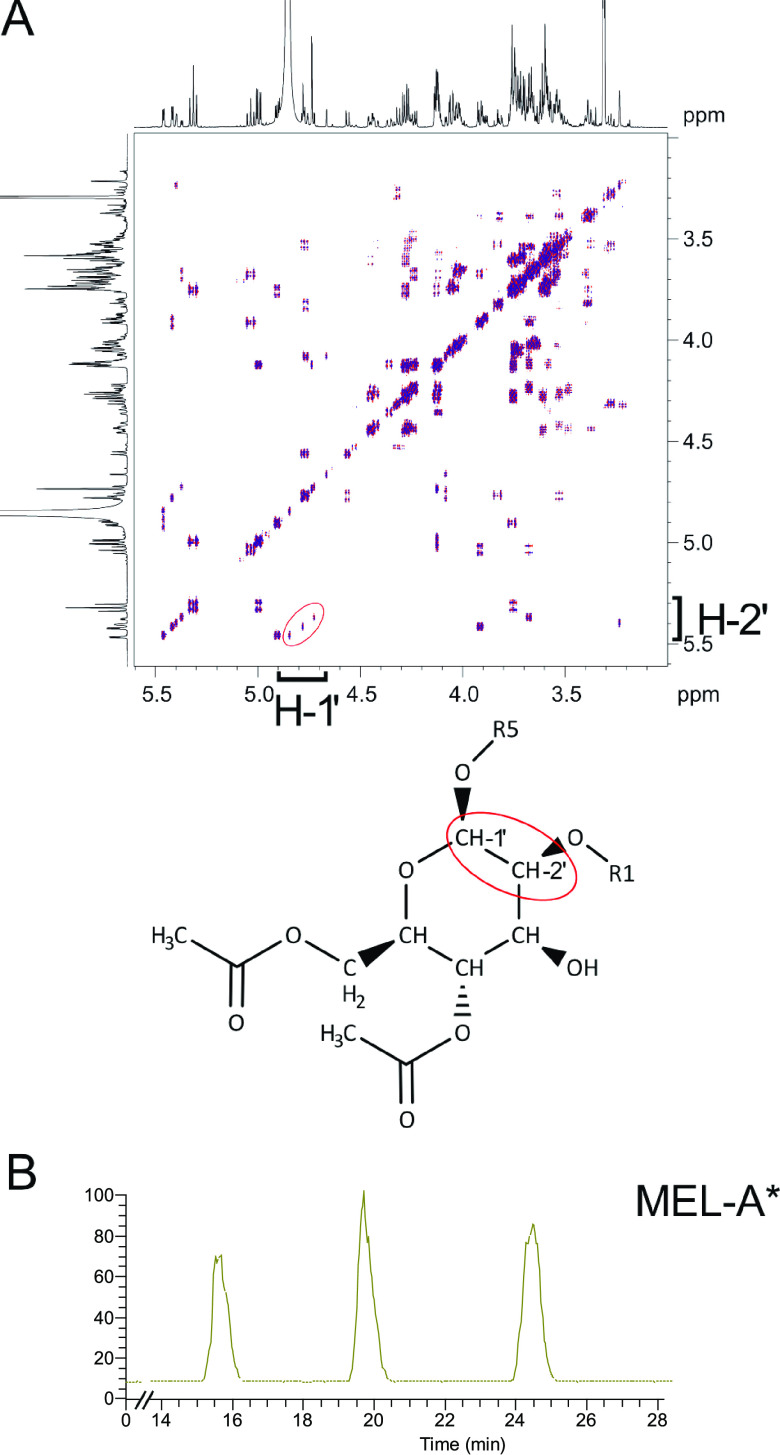
MEL-A* variants contain the acyl side chain at C-2′. (A) Section of the DQF-COSY spectrum of the purified MEL-A* variant in methanol-*d*_4_ at room temperature. Cross peaks indicated in the red circle show the connectivity between H-1′ and H-2′ of the mannose backbone. (B) Total ion counts of MEL-A* variants after purification of the molecules with a mass of 489.1924 *m/z* using the optimized gradient described in Materials and Methods.

To test this concept, we generated a strain lacking both the transporter Mmf1 and the acetyltransferase Mat1. The deletion of *mat1* completely suppressed the phenotype of Δ*mmf1* strains. Nonacetylated MELs were observed in similar amounts in Δ*mat1* and Δ*mat1* Δ*mmf1* cells (Fig. 2A). We again addressed the secretion of MELs by treatment with Tween 20 prior to the extraction of glycolipids from the pellet and supernatant fractions. Nonacetylated MEL-D occurred in the supernatant in the presence as well as in the absence of Mmf1 (Fig. 2B). Thus, Mmf1 is specifically involved in the secretion of molecules harboring acetyl groups but is dispensable for the secretion of MELs without acetyl groups. The unusual MEL variant characteristic for Δ*mmf1* strains was not produced by Δ*mat1* Δ*mmf1* cells, suggesting that it is derived from mature acetylated MELs (Fig. 2A). To obtain detailed insight into the chemical composition of MELs produced by Δ*mmf1* and Δ*mmf1* Δ*mat1* cells, mass spectrometric analyses were performed. Glycolipids isolated from the Δ*mmf1* Δ*mat1* double mutant were highly similar to glycolipids from the Δ*mat1* mutant (Fig. 2C). The unusual MEL produced and secreted by the Δ*mmf1* strain contained only a single acyl group (25). It exhibited a characteristic mass of 489.1924 *m/z* and thus lacked the long side chain normally attached by Mac2 (Fig. 2C). This was confirmed by chromatographic separation and subsequent mass spectrometric analyses (Fig. S4). Monoacylated MEL was absent from glycolipid preparations derived from Δ*mat1* and Δ*mat1* Δ*mmf1* cells. Since the production of MEL-A is a prerequisite for the generation of the unusual molecule, it was termed MEL-A* (Fig. 2C and Fig. S4B). To deduce the structure of the molecule, we analyzed purified MEL-A* by nuclear magnetic resonance (NMR) spectroscopy. The ^1^H spectrum in methanol-*d*_4_ at 27°C showed signals of three different isomers. Due to the severe signal overlap, a full structural characterization was not possible. Nevertheless, the characteristic resonance signals of H-1′, H-2′, C-1′, and C-2′ of the sugar backbone and the connectivity between H-1′ and side chain 1 and between H-2′ and side chain 2 provided unambiguous evidence of an esterification at position 2′ in the sugar (Fig. 3A and Fig. S5). Using a different solvent gradient in the high-performance liquid chromatography (HPLC) experiment, we indeed detected a mixture of three isoforms in MEL-A* preparations (Fig. 3B). These probably resulted from isomerization yielding molecules with acetyl groups at different positions. This shows that MEL-A* consists of three different isoforms, which can spontaneously derive from one another. Together, our data suggest that a malfunction of the export system leads to the deacylation of MEL-A, yielding the MEL-A* variants. In addition, we establish that at least MEL-A can be metabolized by U. maydis.

10.1128/mbio.02123-22.4 FIG S4 Characterization of the novel MEL variant. (A) Enrichment of the unusual MEL variant using silica gel chromatography. The indicated fractions were separated by TLC. The corresponding elution profiles of the mass 489.1924 *m/z* of each fraction are inserted at the bottom. As a control, glycolipids of Δ*mmf1* cells are loaded on the left. (B) MS^2^ fragmentation of the mass 489.1924 *m/z* revealed three main signals at 367.1357 *m/z*, 373.1100 *m/z*, and 429.1719 *m/z*. The corresponding fragmented molecules are depicted. Download FIG S4, PDF file, 0.5 MB. Copyright © 2022 Becker et al. 2022 Becker et al. https://creativecommons.org/licenses/by/4.0/ This content is distributed under the terms of the Creative Commons Attribution 4.0 International license.

10.1128/mbio.02123-22.5 FIG S5 Section of the ^1^H-^13^C HSQC spectrum of the title compound in methanol-*d*_4_ at room temperature. The cross peaks indicated in the red circles are the C-H one-bond correlations of C-1′ and C-2′ of mannose. Download FIG S5, PDF file, 0.2 MB. Copyright © 2022 Becker et al. 2022 Becker et al. https://creativecommons.org/licenses/by/4.0/ This content is distributed under the terms of the Creative Commons Attribution 4.0 International license.

### Feeding assays demonstrate the uptake and modification of MEL-D by U. maydis.

**FIG 4:**
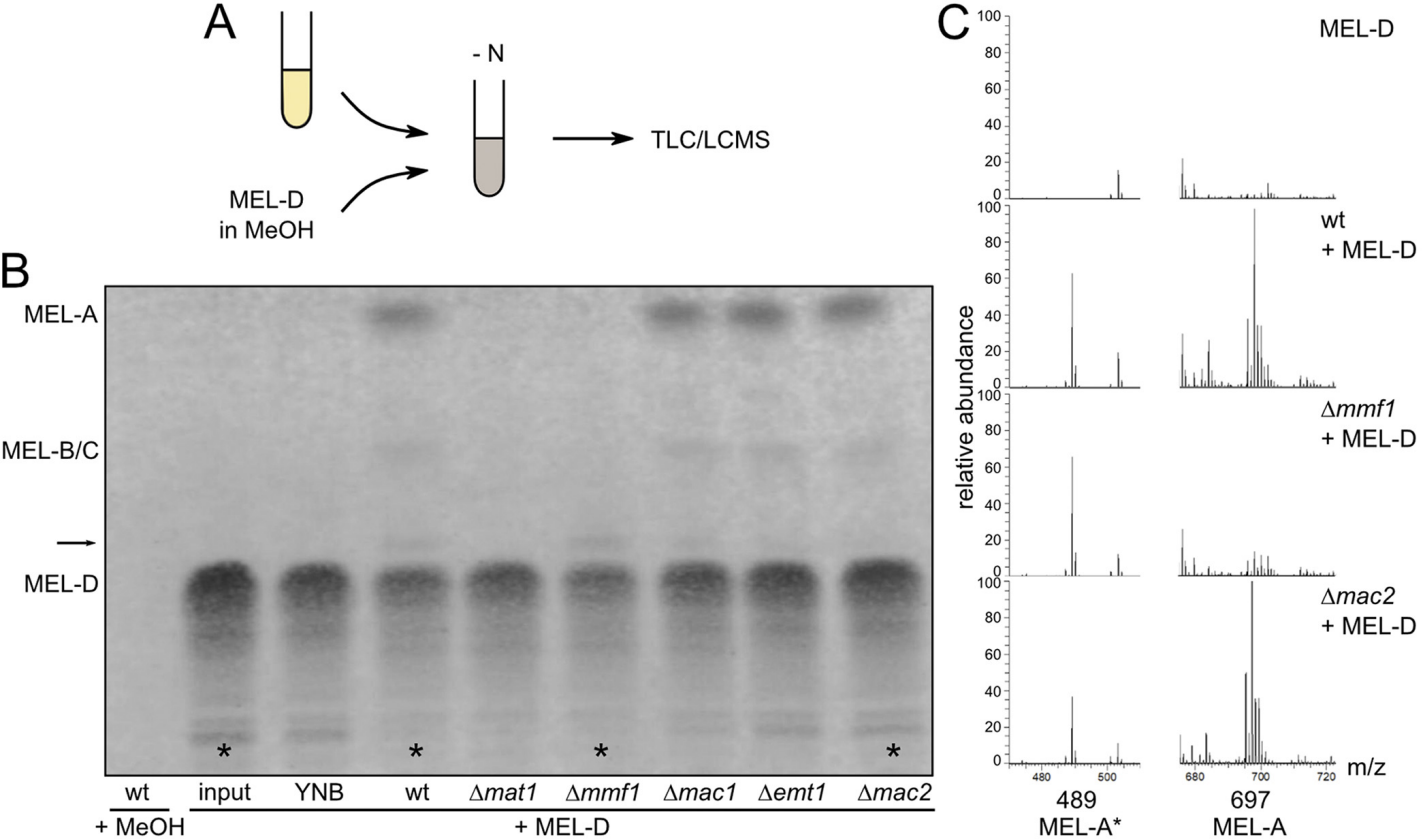
Feeding experiments reveal the uptake and modification of MEL-D. (A) Schematic drawing illustrating the workflow of feeding experiments. Cultures grown overnight were diluted in YNB without carbon and nitrogen sources and fed with either methanol or MEL-D dissolved in methanol. After 6 days, MELs were extracted and analyzed by TLC or LCMS. (B) TLC of feeding experiments with MEL-D in the indicated strains. The arrow highlights the MEL-A* variant. The asteriks mark the MELs analyzed in C. (C) Total ion counts of MELs recovered from the indicated strains. Peaks corresponding to MEL-A* (489.1924 *m/z*) and diacetylated MELs (697.3774 *m/z*) with C_20:1_ at R1 and R2 (e.g., C_4_ plus C_16:1_) are specified.

To further investigate this process, we developed a glycolipid feeding assay (Fig. 4A). We reasoned that the potential uptake and metabolization of purified MELs may allow us to reconstitute the series of reactions for the biosynthesis, secretion, potential uptake, and degradation of MELs and to decipher their function as a putative external carbon source. Thus, cultures of WT, Δ*mat1*, and Δ*mmf1* cells were supplemented with MEL-D prepared from Δ*mat1* cells as the sole carbon source. While in WT and Δ*mmf1* cells, MEL-D was taken up and further metabolized, Δ*mat1* cells produced neither acetylated MELs nor MEL-A* (Fig. 4B). WT cells secreted predominantly diacetylated MEL-A, demonstrating that MELs can be taken up, acetylated, and secreted again. In Δ*mmf1* cells, small amounts of MEL-A* were detected. This strengthens our concept that MEL-A* is generated from MEL-A if export is blocked. To confirm that this assay reflects the uptake and further modification of purified MELs rather than *de novo* biosynthesis, the feeding assay was performed with Δ*emt1*, Δ*mac1*, and Δ*mac2* strains unable to synthesize MELs (10). The accumulation of diacetylated MEL-A and also small amounts of MEL-A* were detected, demonstrating the uptake of MEL-D and further modification through Mat1 (Fig. 4B). Mass spectrometric experiments confirmed these observations (Fig. 4C). Of interest, Δ*mac2* mutants were able to synthesize MEL-A; however, the subsequent production of MEL-A* was reduced. Hence, this feeding experiment verified the uptake and further metabolism of MELs by U. maydis and suggests a role for Mac2 in the degradation of MEL-A to MEL-A*. Degradation of MELs may therefore involve the reversible action of the acyltransferase Mac2 required for MEL biosynthesis.

### Uptake and degradation of acetylated MELs.

**FIG 5:**
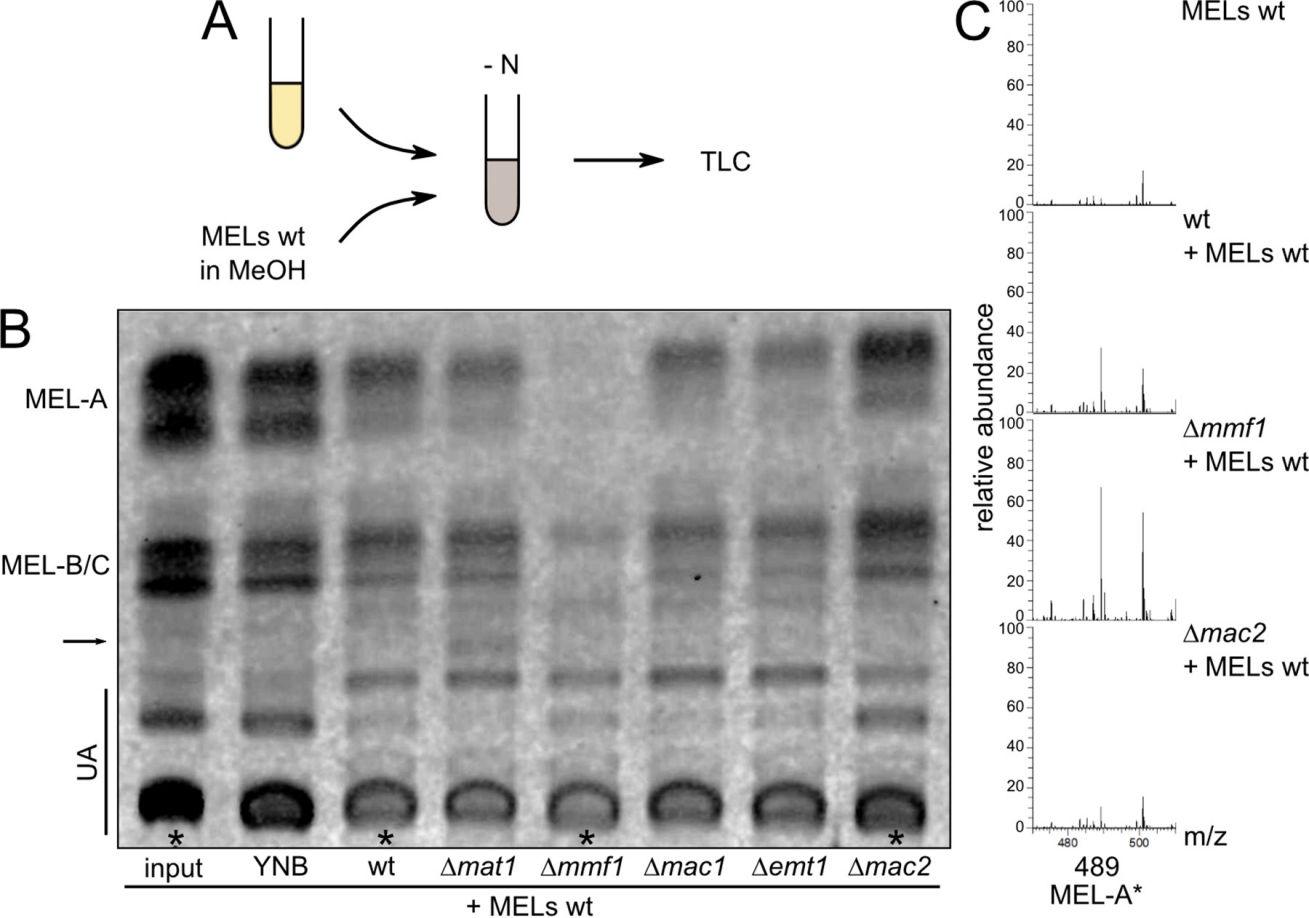
Feeding experiments with acetylated MELs from wild-type cells. (A) Schematic drawing illustrating the workflow of feeding experiments. Cultures grown overnight were diluted in YNB without carbon and nitrogen sources and fed with MELs dissolved in methanol. After 6 days, MELs were extracted and analyzed by TLC or LCMS. (B) TLC of feeding experiments with acetylated MELs in the indicated strains. The arrow highlights the MEL-A* variant. The asteriks mark the MELs analyzed in C. (C) Total ion count spectra of the MELs recovered from the indicated strains. Peaks correspond to MEL-A* (489.1924 *m/z*).

Next, we performed a similar set of assays focusing on the uptake and catabolism of acetylated MELs (Fig. 5A) and added preparations consisting mainly of MEL-A, MEL-B, and MEL-C to the WT or Δ*mat1* and Δ*mmf1* mutants but also to mutants that are unable to synthesize MELs (Δ*emt1*, Δ*mac1*, and Δ*mac2*). We observed significant uptake and depletion of MELs not only in WT cells but also in Δ*mmf1*, Δ*mat1*, Δ*mac1*, and Δ*emt1* cells (Fig. 5B). Remarkably, MEL-A* was produced upon incubation of Δ*mat1* cells with preparations of acetylated MELs (Fig. 5C). These results substantiate the following series of events for glycolipid biosynthesis and secretion. First, the carbohydrate backbone is acylated by Mac1 and Mac2. Next, Mat1-catalyzed acetylation occurs, and acetylated MELs are finally exported by Mmf1. Although incapable of export, Δ*mmf1* mutants can import and degrade acetylated MELs, demonstrating that the protein serves as a specific exporter for these molecules but is dispensable for import. Furthermore, a decrease in MELs was detected in WT, Δ*mat1*, Δ*mmf1*, Δ*mac1*, and Δ*emt1* cells but not in mutants lacking the acyltransferase Mac2 (Fig. 5B). This again indicates that Mac2 may be directly involved in the breakdown of glycolipids.

Together, our results show that U. maydis can catabolize MELs, suggesting a biological function of MELs as an external carbon supply. In theory, this may allow the mutual support of U. maydis cells inside populations.

### Exchange of MELs between cells in cocultivation.

**FIG 6:**
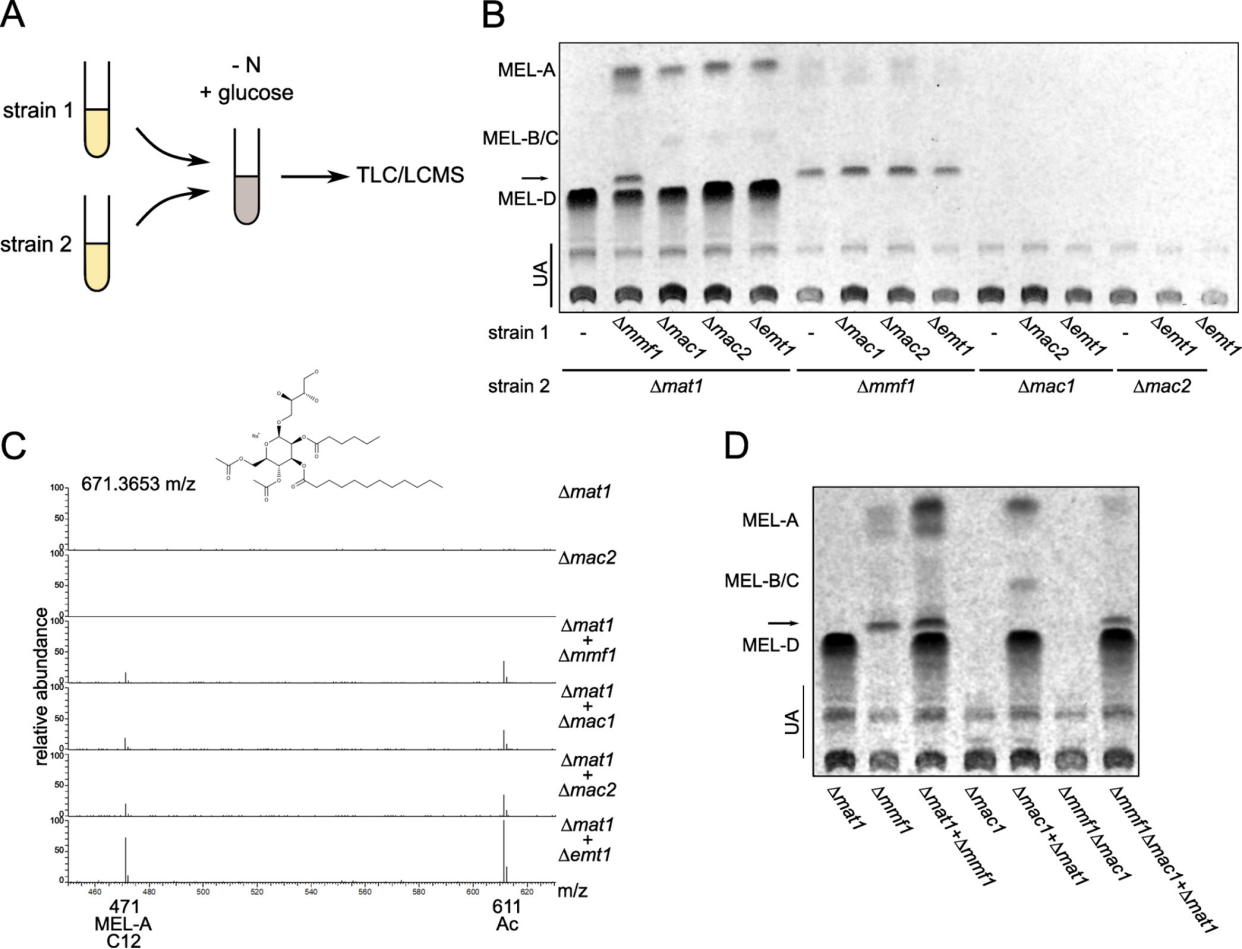
Exchange of MELs upon cocultivation. (A) Schematic drawing of the cocultivation setup. The indicated individual strains were grown overnight. Strains were diluted to an OD at 600 nm (OD_600_) of 0.05 in YNB plus 1% glucose and either mixed or grown in isolation. After 6 days, MELs were extracted and analyzed by TLC. (B) TLC of MELs extracted from cocultivations of the indicated deletion mutants. The arrow marks the MEL-A* variant. (C) MS^2^ analysis of the mass of 671.3653 *m/z* characteristic of diacetylated MELs with a total of 18 C atoms acylated at R1 and R2 (Fig. 1A). Fragmentation products correspond to monoacetylated variants (611.3442 *m/z*, Δ60.0211 *m/z*; acetic acid) and the monoacylated variant (471.1877 *m/z*, Δ200.1776 *m/z*; C_12_ fatty acid). (D) TLC of cocultivations of the indicated single- and double-deletion strains. The arrow marks the MEL-A* variant.

Since our experiments hint at a role for MELs as a potential carbon source for U. maydis, we asked if the glycolipids can be exchanged between cocultivated mutants. Hence, we incubated the Δ*mat1* and Δ*mmf1* strains together with other MEL biosynthesis mutants in the absence of nitrogen to induce MEL biosynthesis and assess the exchange of molecules (Fig. 6A). We observed significant uptake and modification of MELs by recipient strains that are unable to synthesize particular molecules due to their genetic background (Fig. 6B). Tandem mass spectrometric analysis revealed that MELs are modified according to the pathway suggested above (Fig. 6C). Deacetylated MEL-D is taken up by recipient strains and acetylated via Mat1, giving rise to MEL-A (Fig. 6C). Furthermore, this assay shows that MEL-A* variants secreted by Δ*mmf1* remained unmodified and were not depleted (Fig. 6B). They may fail to be imported or cannot be further modified. We also mixed Δ*mat1* mutants with Δ*mmf1* Δ*mac1* double mutants that are unable to generate MELs on their own and observed the production of MEL-A* molecules (Fig. 6D).

Together, these experiments demonstrate the exchange of molecules and their modification and degradation in cocultivation experiments.

## DISCUSSION

**FIG 7:**
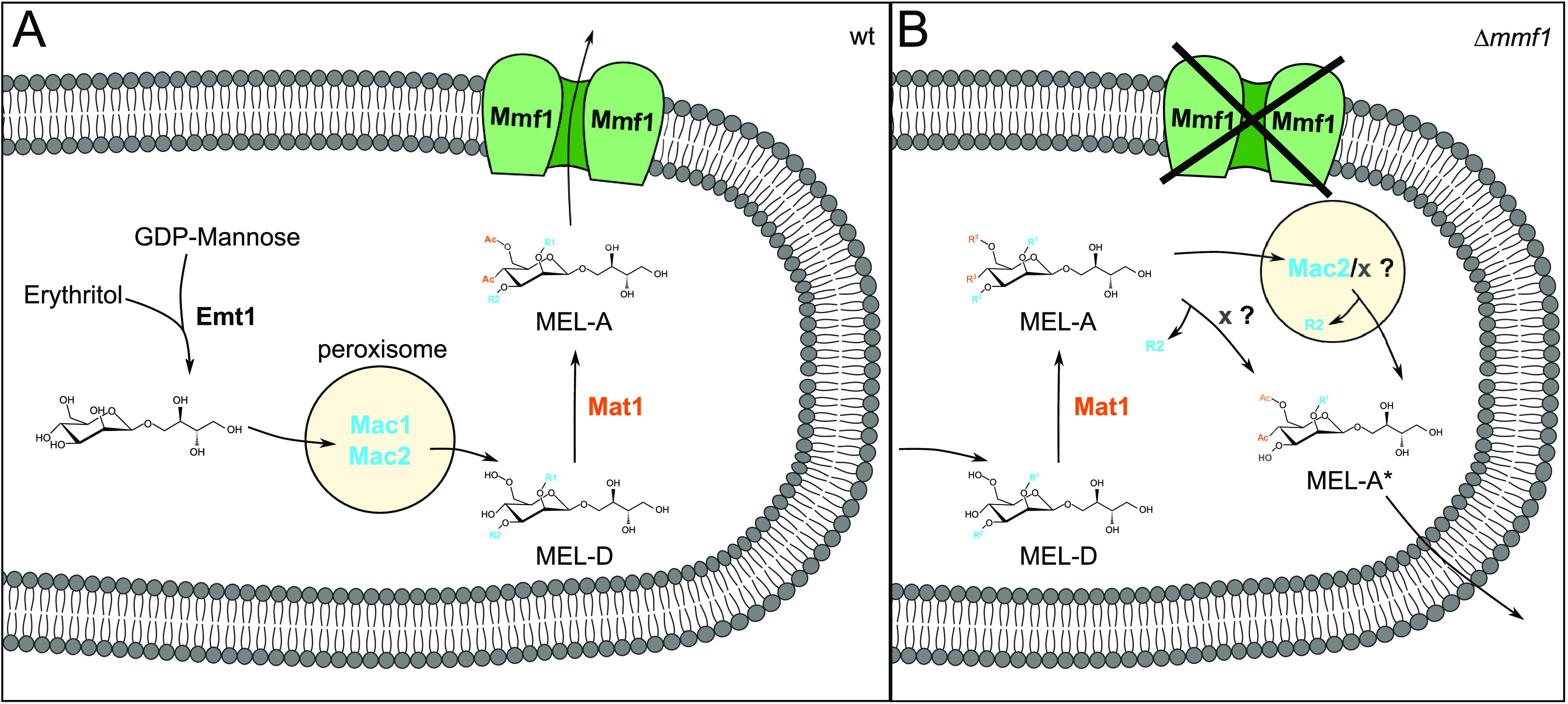
Model for MEL biosynthesis and export. The Mmf1 transporter is critical for the export of acetylated MEL variants. Acetylated MELs are degraded to MEL-A* and exported in this modified form when Mmf1 is lacking.

Our study clarified the order of events during the biosynthesis of MELs in the smut fungus U. maydis. The combination of mutant analyses and feeding experiments revealed that the carbohydrate backbone of MELs is first acylated, probably inside peroxisomes (11). Acylated MELs subsequently acetylated at the plasma membrane and exported by Mmf1 in its acetylated state (Fig. 7A). If the transport protein Mmf1 is absent, acetylated MELs fail to be exported and are degraded via the monoacylated MEL-A* molecules, which can be secreted independently of Mmf1 (Fig. 7B). Nonacetylated MEL-D is exported out of the cell in the absence of Mmf1. Our results highlight the value of the applied combination of feeding and cocultivation experiments with a defined set of mutants as an approach to characterize the biosynthetic pathway and the route of transport of a secondary metabolite.

We have uncovered the function of Mmf1 as a transport protein specific for acetylated MELs. This suggests similar functions of the transport proteins in U. maydis and P. tsukubaensis, albeit diacetylated MELs are not produced by this fungus (29, 30). Exact knowledge about the pathway for MEL synthesis and transport is key to further improve MEL biosynthesis for biotechnology and to harness fungal strains for the efficient production and secretion of biosurfactants (7, 15, 31–33).

Besides this major insight into the biosynthetic pathway for MELs, our work provides evidence for an unexpected biological role. We demonstrate that MELs can be taken up and metabolized by U. maydis cells. We were surprised by the observation that strains lacking Mac2 are less effective in metabolizing MELs. Mac2 is not absolutely essential for the degradation of MELs since small amounts of MEL-A* variants were still observed in Δ*mac2* cells (Fig. 5). Nevertheless, our data suggest a reversible reaction.

Many different biological functions of secondary metabolites are well characterized (1), but probably even more await elucidation. The finding that U. maydis cells are capable of exchanging MELs opens a novel avenue to assess the biological relevance of glycolipids. So far, MELs have been known for reducing surface tension and antimicrobial activity and, hence, may neutralize competitors (34). They were previously suggested to be a potential external carbon source and a solvent to facilitate the uptake of limiting nutrients (3, 35). Here, we demonstrate that MELs can be exchanged and metabolized by Ustilago maydis cells and can indeed be regarded as external carbon storage. Their exchange may enable mutual support inside a population of cells. It was shown previously that the expression of the gene cluster for MEL biosynthesis is significantly upregulated on the plant surface prior to infection (36, 37). It is tempting to speculate that MELs have a role in the early biotrophic development of U. maydis (38), possibly as an external carbon source for fungal hyphae in a better location for infection. This may be important under more natural conditions of infection since mutants defective in MEL biosynthesis are able to induce tumors when large amounts of cells are inoculated into maize plants (39). As MELs are probably mobile on the hydrophobic surface of the plant, they have the potential to spread and cover some distance.

## MATERIALS AND METHODS

### Strains and growth conditions.

U. maydis strains were grown at 28°C in liquid YEPS (YEPS-L) medium (1% yeast extract, 0.4% peptone, 0.4% sucrose) or on solid potato dextrose broth containing 1.5% Bacto agar. All U. maydis strains used and generated in this study are listed in Table S1 in the supplemental material and are derivatives of the U. maydis wild-type strain MB215 (39). To induce glycolipid production, U. maydis strains were inoculated into nitrogen starvation medium (optical density [OD] = 0.1) containing 0.17% YNB (yeast nitrogen base without ammonium sulfate) and 1% glucose as a carbon source and grown for 3 to 6 days at 23°C or 30°C. Escherichia coli strain Top10 (Invitrogen) was used for the transformation and amplification of plasmid DNA (40).

10.1128/mbio.02123-22.6 TABLE S1 Strains used in this study. Download Table S1, DOCX file, 0.02 MB. Copyright © 2022 Becker et al. 2022 Becker et al. https://creativecommons.org/licenses/by/4.0/ This content is distributed under the terms of the Creative Commons Attribution 4.0 International license.

### Plasmid and strain construction.

Standard procedures were performed for molecular cloning and DNA analysis (41). The transformation of U. maydis was performed as described previously (42). Genomic DNA of U. maydis cells was prepared according to a protocol described previously by Hoffman and Winston (43). Constructs for gene replacement were generated as established previously, using an SfiI-based cassette system and a PCR-based cloning strategy (44, 45). Plasmids for the complementation of the Δ*mmf1* or Δ*mat1* Δ*mmf1* deletion mutant were based on plasmid petef-Ala6-MMXN (46) or were described previously (47). Linearized plasmids were integrated into the *ip* locus, producing carboxin-resistant strains (48, 49). The *rua1* gene was removed by the CRISPR/Cas9 system using the pSM2 plasmid (50, 51). Primer sequences are listed in Table S2. Detailed cloning procedures are available from the authors. All plasmids listed in Table S3 were verified by sequencing.

10.1128/mbio.02123-22.7 TABLE S2 Primers used in this study. Download Table S2, DOCX file, 0.02 MB. Copyright © 2022 Becker et al. 2022 Becker et al. https://creativecommons.org/licenses/by/4.0/ This content is distributed under the terms of the Creative Commons Attribution 4.0 International license.

10.1128/mbio.02123-22.8 TABLE S3 Plasmids used in this study. Download Table S3, DOCX file, 0.01 MB. Copyright © 2022 Becker et al. 2022 Becker et al. https://creativecommons.org/licenses/by/4.0/ This content is distributed under the terms of the Creative Commons Attribution 4.0 International license.

### Cocultivation.

Cultures of individual strains grown overnight (in YEPS-L medium) were inoculated alone or in a mixture in 3 mL nitrogen starvation medium (OD = 0.05) containing 0.17% YNB and 1% glucose as a carbon source and grown for 6 days at 23°C. Glycolipids were extracted as described previously (10).

### Feeding experiments.

Cultures of individual strains grown overnight (in YEPS-L medium) were inoculated into 3 mL nitrogen starvation medium (OD = 0.1) containing 0.17% YNB and 1% methanol (MeOH) or 1% MEL-A (5 mg/mL) or 1% MEL-D (10 mg/mL) in methanol and grown for 6 days at 23°C. Glycolipids were extracted from the entire culture and dissolved in 30 μL methanol.

### Analysis of glycolipids.

Extracellular glycolipids were extracted as described previously (39). For the secretion analysis, 1 mL of the culture was centrifuged softly (1,500 × *g* for 5 min), and the supernatant was saved. The pellet was washed twice with YNB and resuspended in 1 mL YNB (pellet fraction [P]). The supernatant was centrifuged (17,000 × *g* for 5 min) and transferred to a new tube (supernatant fraction [SN]). The extraction of glycolipids was performed as described above. Tween 20 was used at a final concentration of 0.05%. Glycolipids were analyzed by thin-layer chromatography (TLC) on silica plates, first with a solvent system consisting of chloroform-methanol-water (65:25:4, vol/vol) for 4 min and then with a second solvent system consisting of chloroform-methanol (9:1, vol/vol) two times for 18 min each (52). For Fig. 2A, only the latter solvent system was used twice. The plates were dried, and sugar-containing compounds were visualized by the application of a mixture of ethanol–sulfuric acid–*p*-anisaldehyde (18:1:1, vol/vol), followed by heating at 150°C for 2 min (53). For the identification of the new MEL variant produced by the Δ*mmf1* mutant, glycolipids from a 25-mL culture were loaded onto a 4-g silica gel with a pore size of 60 Å (Fluka) equilibrated with ethyl acetate. Fractionation was done by the Äkta purifier system (GE Healthcare), using a stepwise gradient of 1%, 2.5%, 7.5%, and 10% methanol in chloroform (5 mL each), followed by 50 mL chloroform-methanol-water (65:25:4). One-milliliter fractions were dried, and the glycolipids were dissolved in 100 μL methanol. Twenty microliters was analyzed by TLC, and 50 μL was used for HPLC-mass spectrometry (MS).

### Mass spectrometry: HPLC.

High-performance liquid chromatography (HPLC) separation of the extracted MELs (50 μL) was performed with an 1100-HPLC system (Agilent) equipped with an EC 125/2 Nucleodur 100-3 C_8_ ec column (Macherey-Nagel, Germany). The gradient, applied at a flow rate of 0.2 mL/min with a column temperature of 45°C, was as follows (buffer A is water with 0.05% formic acid, and buffer B is methanol with 0.045% formic acid): a linear gradient from 60% buffer B to 95% buffer B within 30 min and then holding at 95% buffer B for 10 min. MEL-A* variants were separated using a 125/2 Nucleodur C_8_ ec column (Macherey-Nagel, Germany) at a flow rate of 0.2 mL/min with a column temperature of 60°C, applying an optimized gradient of solvent A (water) and solvent B (MeOH), starting with 30% solvent B, with a linear increase to 50% solvent B within 25 min, followed by a linear increase to 95% solvent B within 5 min and holding at 95% solvent B for an additional 5 min.

### Mass spectrometry: electrospray ionization.

Online electrospray ionization MS and MS*^n^* of the HPLC-separated compounds was done using a Finnigan LTQ-FT Ultra Fourier transform (FT) ion cyclotron resonance (FT-ICR) mass spectrometer (Thermo Fisher). Electrospray ionization parameters were adapted to the flow rate and mass range. Accurate masses (accuracy of 2 ppm or better), allowing the determination of the chemical formulas of the eluting compounds, were obtained by using the FT mass analyzer at a resolution of 100,000. Meanwhile, fragment ions were generated and analyzed in the LTQ mass analyzer. Alternatively, data-dependent fragmentation (untargeted) or fixed *m/z* fragmentation (targeted) was used, whereby the latter resulted in better signal-to-noise ratios and sensitivities. The accurate FT masses in combination with MS^2^ experiments were sufficient to identify the acylation pattern of the compounds. Data were analyzed using Xcalibur software (Thermo Fisher).

### Nuclear magnetic resonance spectroscopy.

The sample was dissolved in 0.6 mL of methanol-*d*_4_ and filled into a 5-mm NMR tube for NMR measurements. NMR spectra were recorded on a Bruker AVII 600 spectrometer at 27°C. The ^1^H,^13^C as well as the two-dimensional correlation spectrum ^1^H-^1^H DQF-COSY (double-quantum-filtered correlation spectroscopy), ^1^H-^13^C heteronuclear single-quantum coherence (HSQC) correlation, and heteronuclear multiple-bond correlation (HMBC) experiments were carried out using standard pulse programs and procedures (54). Chemical shifts are given in parts per million, referring to residual solvent signals.

### Accession numbers and alignments.

Accession numbers were obtained from the National Center for Biotechnology Information (NCBI) (www.ncbi.nlm.nih.gov/). *Ustilago hordei*: Mmf1 (UHOR_04873; CCF52715). *U. maydis*: Mmf1 (UMAG_03115; XP_011389466), UMAG_02598 (XP_011388999), UMAG_05421 (XP_011392110). *Moesziomyces aphidis*: Mmf1 (PaG_03510; ETS61962). *Pseudozyma. tsukubaensis*: Mmf1 (BCG44412). *Moesziomyces antarcticus*: Mmf1 (M9M5N8). *Saccharomyces cerevisiae*: Vba5p (NP_013031). *Caenorhabditis elegans*: Hmit1.2 (NP_507624). *Zea mays*: Stp8 (ONM52330). Transmembrane domains were predicted using Phyre2 (55). For Clustal analyses, the multiple-sequence alignment tool from the Kyoto University Bioinformatic Center was used (56).

### Microscopy.

A total of 200 μL of hot 1.5% agarose melted in water was used to create a thin agarose cushion on a 76- by 26-mm microscope slide (Roth). Cells were washed with water and concentrated 5-fold, and 3 μL was spotted onto the middle of the agarose pad and covered with an 18- by 18-mm coverslip (Roth). Microscopy was performed on an Axiovert 200 M inverse microscope (Zeiss) equipped with a 1394 Orca-ERA charge-coupled-device (CCD) camera (Hamamatsu Photonics), filter sets for enhanced GFP (EGFP) and 4′,6-diamidino-2-phenylindole (DAPI) (Chroma Technology), and a Zeiss 63× Plan Apochromat oil lens objective (numerical aperture [NA], 1.4). Single-plane bright-field or phase-contrast images and z-stacks of the cells (0.5-μm z-spacing) in the appropriate fluorescence channels were recorded using Volocity 5.3 image acquisition software (Perkin-Elmer). Images were processed and evaluated using ImageJ (57).
