# Supplementary figures and images for: Import and Export of Mannosylerythritol Lipids by Ustilago maydis
Source: mBio. 2022 Sep 7;13(5):e02123-22. doi: 10.1128/mbio.02123-22 (PMC9600162; doi:10.1128/mbio.02123-22)

**A**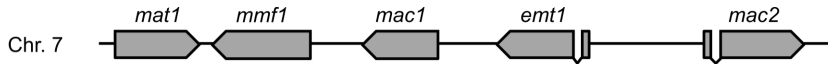**B**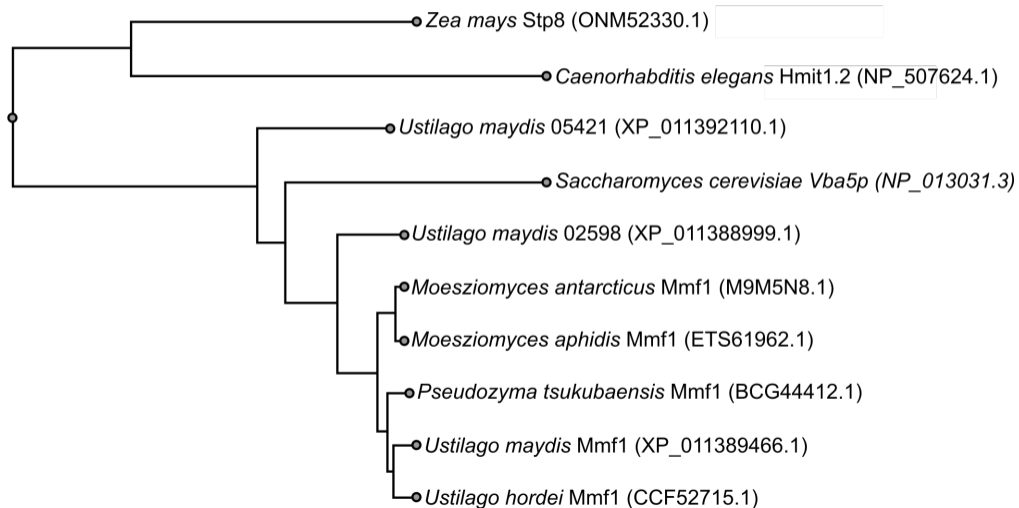

Supplement: FIG S1 [file mbio.02123-22-s0001.pdf]

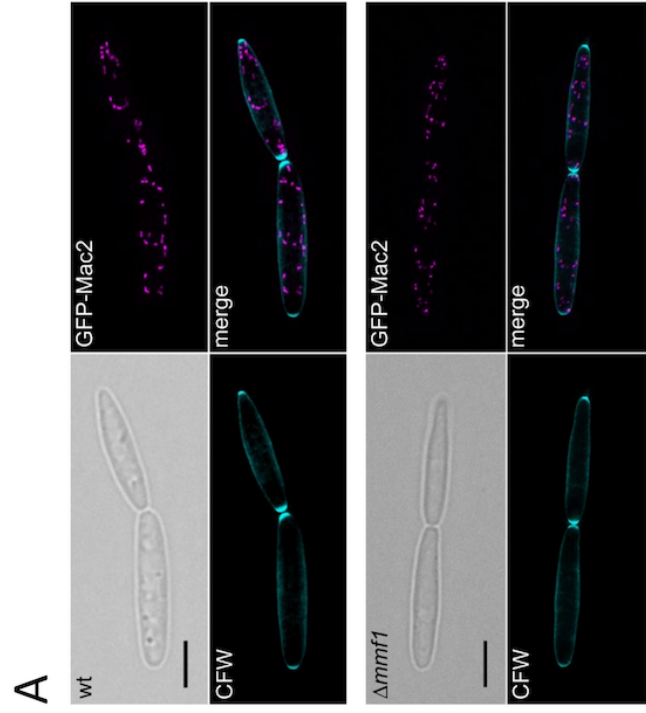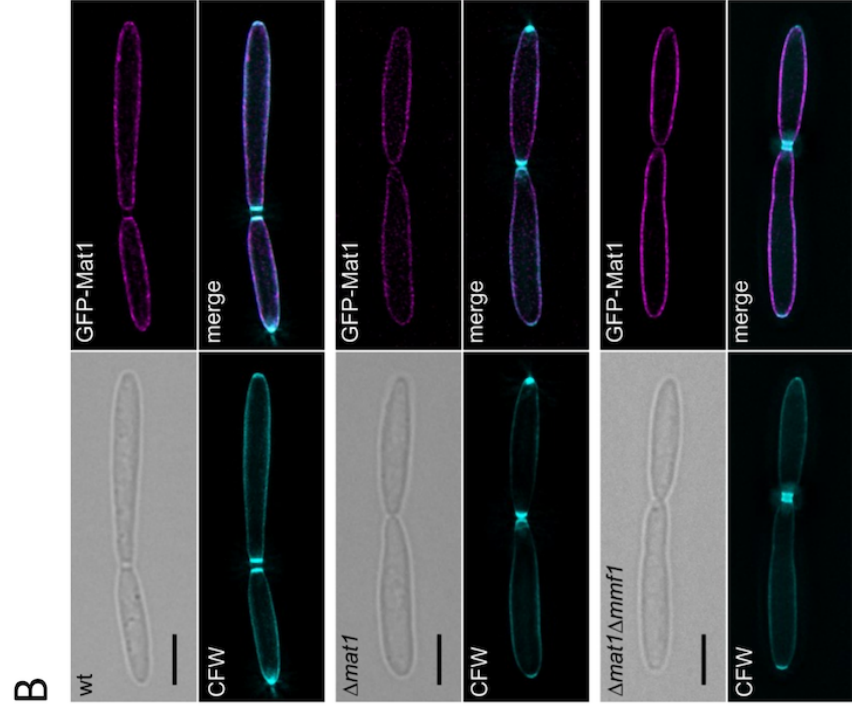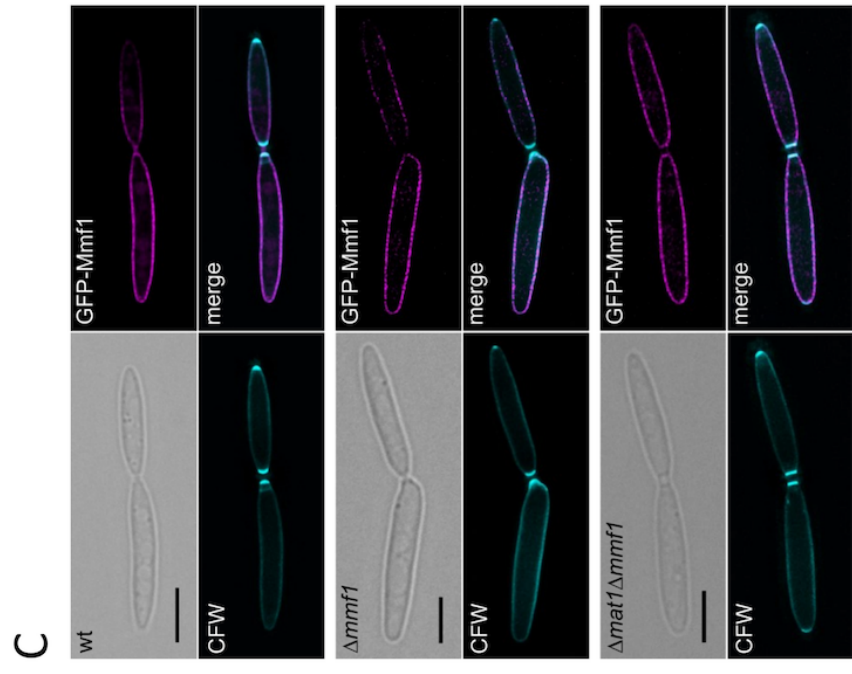

Supplement: FIG S2 [file mbio.02123-22-s0002.pdf]

$\Delta rua1$

$\Delta rua1 \Delta mmf1$

MEL-A

MEL-B/C

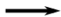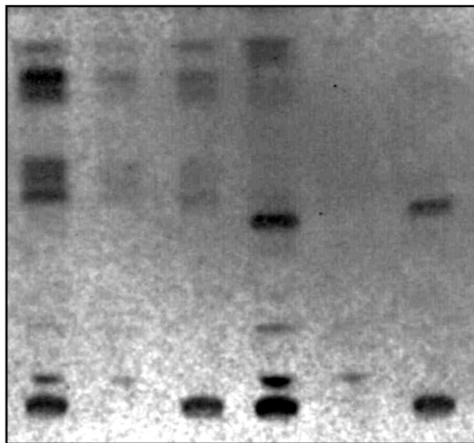

E

P

SN

E

P

SN

Supplement: FIG S3 [file mbio.02123-22-s0003.pdf]

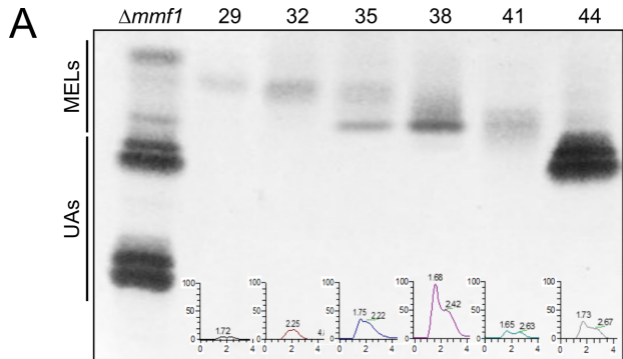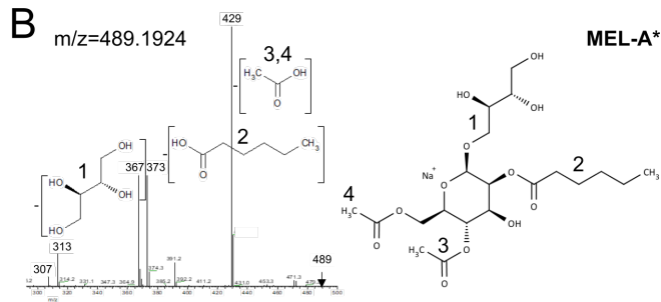

Supplement: FIG S4 [file mbio.02123-22-s0004.pdf]

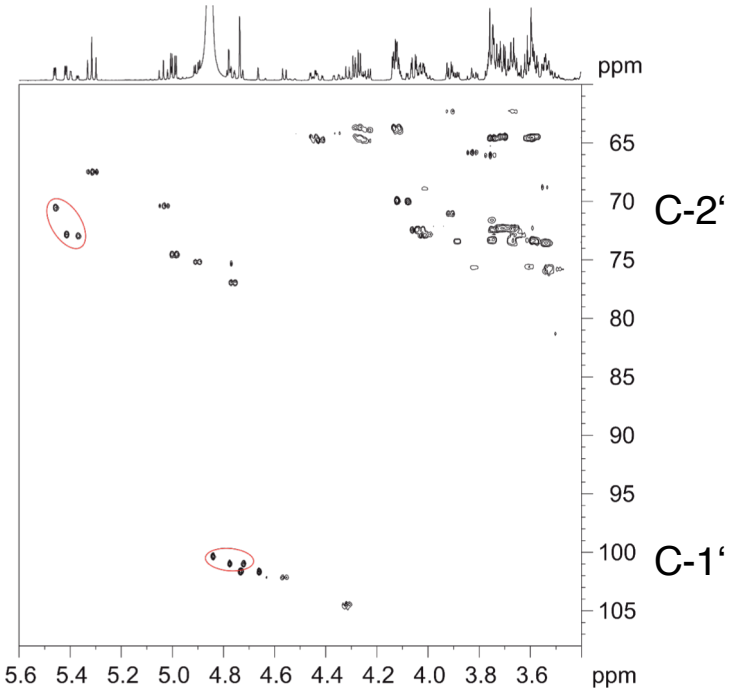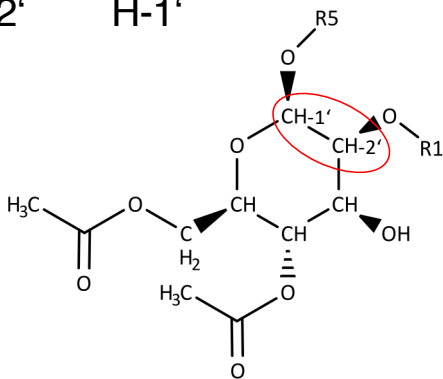

Supplement: FIG S5 [file mbio.02123-22-s0005.pdf]
