# Supplementary material for: Import and Export of Mannosylerythritol Lipids by Ustilago maydis
Source: mBio. 2022 Sep 7;13(5):e02123-22. doi: 10.1128/mbio.02123-22 (PMC9600162; doi:10.1128/mbio.02123-22)
Supplement: TABLE S1 [file mbio.02123-22-s0006.docx]

**Supplementary table 1**

**Strains used in this study**

| Strain | Reference | DNA transformed | Integration | Progenitor strain |
| --- | --- | --- | --- | --- |
| *Ustilago maydis* MB215 | (10) |  |  |  |
| MB215 ∆*mat1* | (11) |  |  |  |
| MB215 ∆*mac2* | (11) |  |  |  |
| MB215 ∆*mmf1* |  | *mmf1*_LF_-hygR-*mmf1*_RF_ |  | MB215 |
| MB215 ∆*mmf1+*  Petef-GFP-Mmf1 |  | pETEF-GFP-Mmf1  (12) | *ip* | MB215 ∆*mmf1* |
| MB215 ∆*mat1*∆*mmf1* |  | *mat1*_LF_-hygR-*mmf1*_LF_ |  | MB215 |
| MB215 ∆*mmf1∆mac1* |  | *mac1*_LF_-NatR-*mac1*_RF_ |  | MB215 ∆*mmf1* |
| MB215 ∆*mac1* | (11) |  |  |  |
| MB215 ∆*emt1* | (10) |  |  |  |
| MB215 ∆*mat1*∆*mmf1 +*  Petef-GFP-Mmf1 |  | pETEF-GFP-Mmf1  (12) | *ip* | MB215 ∆*mat1*∆*mmf1* |
| MB215 ∆*mat1*∆*mmf1 +*  Petef-GFP-Mat1 |  | pETEF-GFP-Mat1  (12) | *ip* | MB215 ∆*mat1*∆*mmf1* |
| MB215 ∆*rua1* |  | pSM2-rua1 |  | MB215 |
| MB215 *∆mmf1∆rua1* |  | pSM2-rua1 |  | MB215 *∆mmf1* |
|  |  |  |  |  |
