# Supplementary material for: Import and Export of Mannosylerythritol Lipids by Ustilago maydis
Source: mBio. 2022 Sep 7;13(5):e02123-22. doi: 10.1128/mbio.02123-22 (PMC9600162; doi:10.1128/mbio.02123-22)
Supplement: TABLE S2 [file mbio.02123-22-s0007.docx]

**Supplemental table S2**

**Primers used in this study**

| Primer | used for | sequence |
| --- | --- | --- |
| MH705 | Mmf1 LF 5’ + SspI | gcaatattGTCTCTTTTGGTTGTAGCTCC |
| MH706 | Mmf1 LF 3’ + SfiI | gtggccatctaggccTGTATACGATCAGTGTACG |
| MH707 | Mmf1 RF 5’ + SfiI | gaggcctgagtggccCACTGCGTGCGCAGCG |
| MH708 | Mmf1 RF 3’ + SspI | gcaatattGATGGCACAAGCTCGCTCGG |
| MC119 | Mat1 LF 5‘ | GATTCCAGGGCGAACTCACGG |
| MC121 | Mat1 LF 3‘ + SfiI | cacggcctgagtggccGATCAGCGACAGCTCGATGTGC |
| MC132 | Mac1 LF 5‘ | CACGACGAGTCAAGTTGTGCCG |
| MC133 | Mac1 LF 3‘ + SfiI | cacggcctgagtggccGCCAAAGAGGTAGATTTGAACC |
| MC134 | Mac1 RF 5‘+ SfiI | gtgggccatctaggccCGCTGTTGGCACTTTACGTTTG |
| MC135 | Mac1 RF 3‘ | GCTGTGGCCTGGTTCAACGTCC |
| MC137 | Mac2 LF 5‘ | GGTTTCAGGCTTCGTGGTCAGG |
| MC139 | Mac2 LF 3‘ + SfiI | cacggcctgagtggccGTTAACCGTGATTTGTGCTACAAC |
| MC140 | Mac2 RF 5‘+ SfiI | gtgggccatctaggccCACTCTGCGACTGTTTTCTG |
| MC141 | Mac2 RF 3‘ | GGAAGTTGGGGACTTTAAGCC |
| MJ815 | Rua1 sgDNA fwd | aacGCGTCGGGCATGCCAGCTGAgttttagag |
| MJ816 | Rua1 sgDNA rev | catgctctaaaacTCAGCTGGCATGCCCGACGCgtt |
|  |  |  |
|  |  |  |
|  |  |  |
|  |  |  |
|  |  |  |
|  |  |  |
|  |  |  |
|  |  |  |
|  |  |  |
|  |  |  |
|  |  |  |
