# Supplementary material for: Import and Export of Mannosylerythritol Lipids by Ustilago maydis
Source: mBio. 2022 Sep 7;13(5):e02123-22. doi: 10.1128/mbio.02123-22 (PMC9600162; doi:10.1128/mbio.02123-22)
Supplement: TABLE S3 [file mbio.02123-22-s0008.docx]

**Supplementary table S3**

**Plasmids used in this study**

| Plasmid | used for | cloning procedure |
| --- | --- | --- |
| pSM2-Rua1 | Deletion of *rua1* | The annealed Primer pair MJ815/MJ816 was cloned in pSM2 digested with SnaBI and XbaI. |
| pETEF-GFP-Mat1 | Complementation of ∆*mat1∆mmf1* | (12) |
| pETEF-GFP-Mmf1 | Complementation of ∆*mmf1*and *∆mat1∆mmf1* | (12) |
|  |  |  |
|  |  |  |
